# Crystal structure of tetra­aquabis(1,3-dimethyl-2,6-dioxo-3,7-di­hydro-1*H*-purin-9-ido)magnesium

**DOI:** 10.1107/S2056989015003758

**Published:** 2015-02-28

**Authors:** Yabin Shi, Benyong Lou

**Affiliations:** a Department of Chemistry and Chemical Engineering, Minjiang University, Fuzhou 350108, People’s Republic of China

**Keywords:** crystal structure, theophylline, tetra­aqua mononuclear Mg^II^ complex, hydrogen bonding

## Abstract

The title complex, [Mg(C_7_H_7_N_4_O_2_)_2_(H_2_O)_4_], lies across an inversion centre and the Mg^II^ atom is coordinated in a slightly distorted octa­hedral environment by four aqua ligands in the equatorial sites and two 1,3-dimethyl-2,6-dioxo-3,7-di­hydro-1*H*-purin-9-ide ligands, through imidazole ring N atoms, in the axial sites. An intra­molecular O—H⋯O hydrogen bond forms an *S*(7) graph-set motif. In the crystal, O—H⋯O and O—H⋯N hydrogen bonds link complex mol­ecules forming a three-dimensional network incorporating *R*_4_^2^(8) and *R*_2_^2^(18) graph-set motifs.

## Abstract 2

The title complex, [Mg(C_7_H_7_N_4_O_2_)_2_(H_2_O)_4_], lies across an inversion centre and the Mg^II^ atom is coordinated in a slightly distorted octa­hedral environment by four aqua ligands in the equatorial sites and imidazole ring N atoms in the axial sites. In the crystal, O—H⋯O and O—H⋯N hydrogen bonds link complex mol­ecules, forming a three-dimensional network incorporating 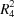
(8) and 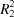
(18) graph-set motifs.

## Chemical context   

Co-crystallization represents a crystal engineering approach for modifying properties of active pharmaceutical ingredients (APIs) (Sun, 2013 ▸). Metal coordination is an alternative strategy without changing chemical structures of APIs (Ma & Moulton, 2007 ▸). Theophylline is a methylxanthine drug in the treatment of asthma and chronic obstructive pulmonary disease (Barnes, 2003 ▸). In this study, we reacted theophylline with the Mg^II^ ion in a basic solution to give rise to a tetra­aqua mononuclear Mg^II^ complex, (I).
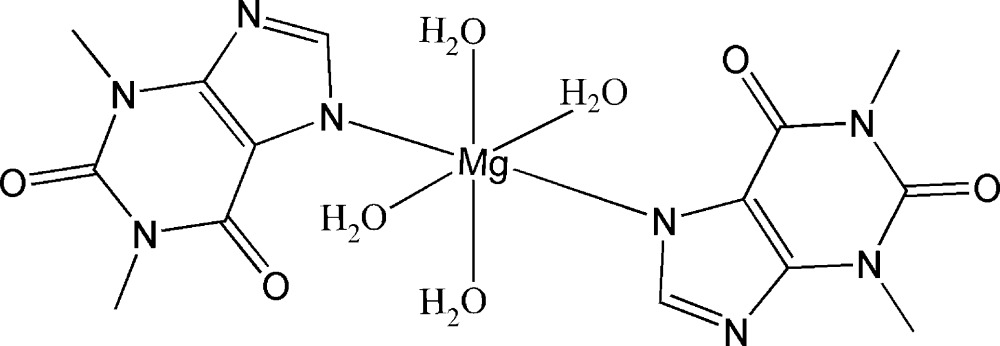


## Structural commentary   

**Figure 1:**
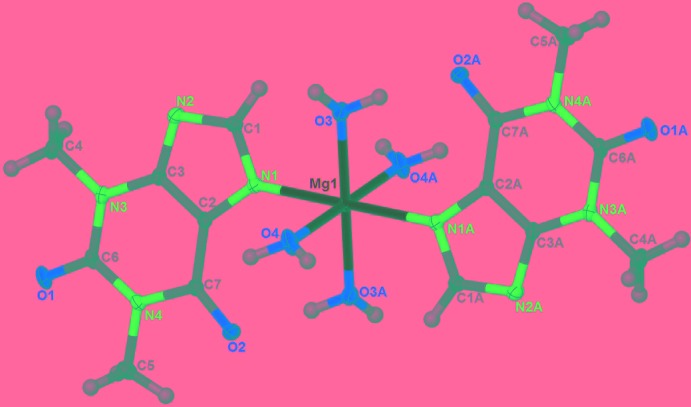
The mol­ecular structure of the title complex, shown with 30% probability displacement ellipsoids [symmetry code: (A) *x*, −*y*, −*z* + 1).

The mol­ecular structure of (I) is shown in Fig. 1 ▸. The complex lies across an inversion centre and the Mg^II^ atom is coordin­ated in a slightly distorted octa­hedral environment (Table 1 ▸) by four aqua ligands in the equatorial sites and two 1,3-dimethyl-2,6-dioxo-3,7-di­hydro-1*H*-purin-9-ide ligands, through imidazole ring N atoms [N1 and N1(−*x* + 1, −*y*, −*z* + 1)], in the axial sites. The symmetry-unique purine ring system is essentially planar, with a maximum deviation of 0.030 (2) Å for N3 and the bonded methyl C atoms C4 and C5 deviate from this mean plane by −0.118 (3) and 0.136 (2) Å, respectively.

## Supra­molecular features   

**Figure 2:**
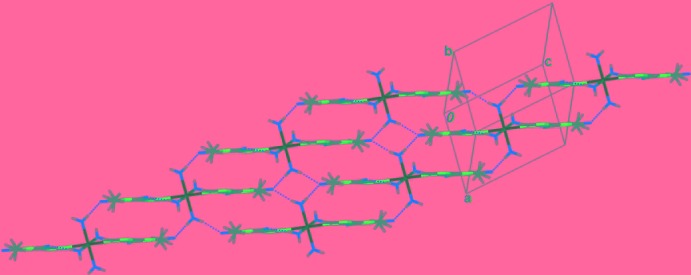
Part of the crystal structure, showing hydrogen bonds in two dimensions (dashed lines).

**Figure 3:**
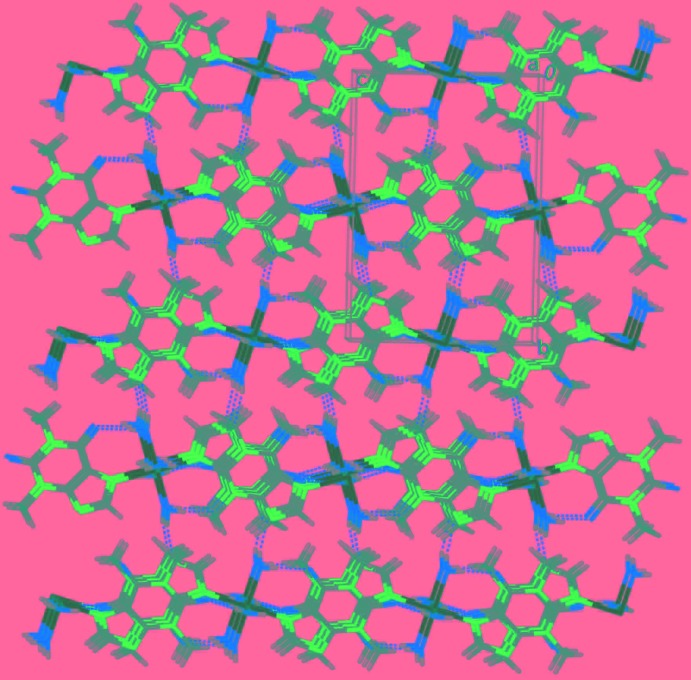
Part of the crystal structure, showing the overall three-dimensional hydrogen-bonded structure (dashed lines).

In the crystal, the coordinating water mol­ecules are involved in various hydrogen-bonding inter­actions (Table 2 ▸). A 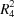
(8) graph-set motif (Bernstein *et al.*, 1995 ▸) is formed through [O4⋯O1^iii^ = 2.829 (3) Å and O4⋯O1^iv^ = 2.780 (2) Å; symmetry codes: (iii) −*x*, −*y*, −*z*; (iv) *x*, *y*, *z* + 1] between a coordinating water mol­ecule and a carbonyl group of a symmetry-related theophylline group. The mononuclear units are connected into a layer parallel to (010) (Fig. 2 ▸), which is further connected into a three-dimensional structure (Fig. 3 ▸) by hydrogen-bonding inter­actions between coordin­ating water mol­ecules and symmetry-related imidazole groups [O3⋯N2^ii^ = 2.809 (3) Å; symmetry code: (ii) *x*, −*y* + 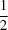, *z* + 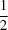].

## Database survey   

A search of the Cambridge Structural Database (Version 5.36, November 2014; Groom & Allen, 2014 ▸) revealed 16 metal complexes of theophylline, including ternary, polynuclear complexes and coordination polymers but only five are mononuclear complexes. The most closely related compound to the title complex, in terms of the ligand types is tri­aqua­bis­(theophylline)copper(II) dihydrate (WEZYIJ; Begum & Manohar, 1994 ▸). The title compound is the first crystal structure reported to date of a complex of theophylline with an alkaline-earth metal.

## Synthesis and crystallization   

Theophylline (180 mg, 1 mmol) was dissolved in water (20 ml). An aqueous solution (15 ml) of NaOH (40 mg, 1 mmol) was added slowly. MgCl_2_·6H_2_O (102 mg, 0.5 mmol) in water (15 ml) was then added. The resulting solution was kept in air and, after several days, colourless block-shaped crystals were obtained.

## Refinement   

Crystal data, data collection and structure refinement details are summarized in Table 3 ▸. H atoms bonded to C atoms were positioned geometrically (C—H = 0.95–0.98 Å) with *U*_iso_(H) = 1.2*U*_eq_(C) or 1.5*U*_eq_(C). H atoms bonded to O atoms were located in difference Fourier maps and were refined with a distance restraint of O—H = 0.87 (1) Å. The isotropic displacement parameters were refined freely.

## Supplementary Material

Crystal structure: contains datablock(s) I, LOU. DOI: 10.1107/S2056989015003758/lh5752sup1.cif

Structure factors: contains datablock(s) I. DOI: 10.1107/S2056989015003758/lh5752Isup2.hkl

CCDC reference: 1050901

Additional supporting information:  crystallographic information; 3D view; checkCIF report

## Figures and Tables

**Table 1:** Selected geometric parameters (, )

Mg1O3^i^	2.0672(17)	Mg1N1^i^	2.2255(19)
Mg1O4^i^	2.081(2)		
			
O3^i^Mg1O3	180.00(3)	O4Mg1N1^i^	91.31(6)
O3Mg1O4^i^	87.98(7)	O3Mg1N1	90.06(6)
O3Mg1O4	92.02(7)	O4Mg1N1	88.69(6)
O4^i^Mg1O4	180.0	N1^i^Mg1N1	180.0
O3Mg1N1^i^	89.94(6)		

Symmetry code: (i) 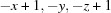.

**Table 2:** Hydrogen-bond geometry (, )

*D*H*A*	*D*H	H*A*	*D* *A*	*D*H*A*
O3H3*A*N2^ii^	0.88(1)	1.97(1)	2.809(3)	160(3)
O3H3*B*O2^i^	0.87(1)	1.80(1)	2.668(2)	173(3)
O4H4*E*O1^iii^	0.87(1)	1.93(1)	2.780(2)	168(3)
O4H4*D*O1^iv^	0.87(1)	1.96(1)	2.829(3)	178(3)

Symmetry codes: (i) 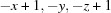; (ii) 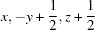; (iii) 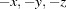; (iv) 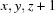.

**Table 3:** Experimental details

Crystal data	
Chemical formula	[Mg(C_7_H_7_N_4_O_2_)_2_(H_2_O)_4_]
*M*_r_	454.71
Crystal system, space group	Monoclinic, *P*2_1_/*c*
Temperature (K)	295
*a*, *b*, *c* ()	7.694(4), 13.399(7), 9.739(5)
()	105.169(9)
*V* (^3^)	969.0(9)
*Z*	2
Radiation type	Mo *K*
(mm^1^)	0.16
Crystal size (mm)	0.2 0.2 0.2

Data collection	
Diffractometer	Rigaku CCD
Absorption correction	Multi-scan (*CrystalClear*; Rigaku, 2000 ▸)
*T*_min_, *T*_max_	0.949, 1.000
No. of measured, independent and observed [*I* > 2(*I*)] reflections	7442, 2153, 1738
*R*_int_	0.032
(sin /)_max_ (^1^)	0.650

Refinement	
*R*[*F* ^2^ > 2(*F* ^2^)], *wR*(*F* ^2^), *S*	0.049, 0.123, 1.09
No. of reflections	2153
No. of parameters	160
No. of restraints	4
H-atom treatment	H atoms treated by a mixture of independent and constrained refinement
_max_, _min_ (e ^3^)	0.29, 0.25

Computer programs: *CrystalClear* (Rigaku, 2000 ▸), *SHELXS97* and *SHELXL97* (Sheldrick, 2008 ▸) and *X-SEED* (Barbour, 2001 ▸).

## supplementary crystallographic information

### Crystal data

**Table:**

[Mg(C_7_H_7_N_4_O_2_)_2_(H_2_O)_4_]	*F*(000) = 476
*M**_r_* = 454.71	*D*_x_ = 1.558 Mg m^−^^3^
Monoclinic, *P*2_1_/*c*	Mo *K*α radiation, λ = 0.71073 Å
Hall symbol: -P 2ybc	Cell parameters from 2170 reflections
*a* = 7.694 (4) Å	θ = 2.7–27.5°
*b* = 13.399 (7) Å	µ = 0.16 mm^−^^1^
*c* = 9.739 (5) Å	*T* = 295 K
β = 105.169 (9)°	Prism, colorless
*V* = 969.0 (9) Å^3^	0.2 × 0.2 × 0.2 mm
*Z* = 2	

### Data collection

**Table:**

Rigaku CCD diffractometer	2153 independent reflections
Radiation source: fine-focus sealed tube	1738 reflections with *I* > 2σ(*I*)
Graphite monochromator	*R*_int_ = 0.032
Detector resolution: 14.6306 pixels mm^-1^	θ_max_ = 27.5°, θ_min_ = 2.7°
CCD_Profile_fitting scans	*h* = −8→9
Absorption correction: multi-scan (*CrystalClear*; Rigaku, 2000)	*k* = −17→17
*T*_min_ = 0.949, *T*_max_ = 1.000	*l* = −11→12
7442 measured reflections	

### Refinement

**Table:**

Refinement on *F*^2^	Primary atom site location: structure-invariant direct methods
Least-squares matrix: full	Secondary atom site location: difference Fourier map
*R*[*F*^2^ > 2σ(*F*^2^)] = 0.049	Hydrogen site location: inferred from neighbouring sites
*wR*(*F*^2^) = 0.123	H atoms treated by a mixture of independent and constrained refinement
*S* = 1.09	*w* = 1/[σ^2^(*F*_o_^2^) + (0.0542*P*)^2^ + 0.4099*P*] where *P* = (*F*_o_^2^ + 2*F*_c_^2^)/3
2153 reflections	(Δ/σ)_max_ < 0.001
160 parameters	Δρ_max_ = 0.29 e Å^−^^3^
4 restraints	Δρ_min_ = −0.25 e Å^−^^3^

### Special details

**Table:**

Geometry. All e.s.d.'s (except the e.s.d. in the dihedral angle between two l.s. planes) are estimated using the full covariance matrix. The cell e.s.d.'s are taken into account individually in the estimation of e.s.d.'s in distances, angles and torsion angles; correlations between e.s.d.'s in cell parameters are only used when they are defined by crystal symmetry. An approximate (isotropic) treatment of cell e.s.d.'s is used for estimating e.s.d.'s involving l.s. planes.
Refinement. Refinement of *F*^2^ against ALL reflections. The weighted *R*-factor *wR* and goodness of fit *S* are based on *F*^2^, conventional *R*-factors *R* are based on *F*, with *F* set to zero for negative *F*^2^. The threshold expression of *F*^2^ > σ(*F*^2^) is used only for calculating *R*-factors(gt) *etc*. and is not relevant to the choice of reflections for refinement. *R*-factors based on *F*^2^ are statistically about twice as large as those based on *F*, and *R*- factors based on ALL data will be even larger.

### Fractional atomic coordinates and isotropic or equivalent isotropic displacement parameters (Å^2^)

**Table:**

	*x*	*y*	*z*	*U*_iso_*/*U*_eq_	
Mg1	0.5000	0.0000	0.5000	0.0220 (2)	
O1	0.0856 (2)	−0.02565 (12)	−0.27579 (14)	0.0366 (4)	
O2	0.2118 (2)	−0.14692 (11)	0.17770 (15)	0.0396 (4)	
O3	0.5654 (2)	0.14587 (11)	0.56135 (16)	0.0361 (4)	
O4	0.2306 (2)	0.02272 (14)	0.49391 (16)	0.0419 (4)	
N1	0.4375 (2)	0.04557 (11)	0.27257 (16)	0.0253 (4)	
N2	0.4425 (2)	0.16133 (12)	0.09955 (17)	0.0295 (4)	
N3	0.2518 (2)	0.07368 (12)	−0.10112 (17)	0.0277 (4)	
N4	0.1533 (2)	−0.08355 (12)	−0.04781 (17)	0.0260 (4)	
C1	0.4967 (3)	0.13335 (14)	0.2386 (2)	0.0285 (4)	
H1	0.5733	0.1745	0.3083	0.034*	
C2	0.3344 (2)	0.01127 (14)	0.14086 (19)	0.0231 (4)	
C3	0.3417 (3)	0.08329 (14)	0.0411 (2)	0.0241 (4)	
C4	0.2540 (4)	0.15494 (17)	−0.2003 (2)	0.0426 (6)	
H4A	0.3623	0.1497	−0.2350	0.064*	
H4B	0.2543	0.2190	−0.1517	0.064*	
H4C	0.1468	0.1507	−0.2809	0.064*	
C5	0.0602 (3)	−0.17694 (16)	−0.1029 (2)	0.0379 (5)	
H5A	−0.0695	−0.1645	−0.1367	0.057*	
H5B	0.0827	−0.2270	−0.0269	0.057*	
H5C	0.1054	−0.2016	−0.1819	0.057*	
C6	0.1602 (2)	−0.01192 (15)	−0.1483 (2)	0.0263 (4)	
C7	0.2344 (3)	−0.07757 (14)	0.1001 (2)	0.0256 (4)	
H3A	0.505 (3)	0.2015 (13)	0.557 (3)	0.058 (8)*	
H3B	0.644 (3)	0.149 (2)	0.6439 (17)	0.059 (9)*	
H4D	0.188 (4)	0.007 (2)	0.566 (2)	0.072 (10)*	
H4E	0.142 (3)	0.023 (2)	0.4175 (19)	0.055 (8)*	

### Atomic displacement parameters (Å^2^)

**Table:**

	*U*^11^	*U*^22^	*U*^33^	*U*^12^	*U*^13^	*U*^23^
Mg1	0.0247 (5)	0.0223 (4)	0.0169 (5)	0.0003 (3)	0.0018 (3)	0.0002 (3)
O1	0.0312 (8)	0.0563 (10)	0.0181 (7)	−0.0009 (7)	−0.0013 (6)	−0.0023 (6)
O2	0.0501 (9)	0.0329 (8)	0.0287 (8)	−0.0141 (7)	−0.0021 (7)	0.0066 (6)
O3	0.0495 (10)	0.0243 (7)	0.0278 (8)	0.0015 (7)	−0.0020 (7)	−0.0018 (6)
O4	0.0278 (8)	0.0722 (12)	0.0239 (9)	0.0027 (8)	0.0034 (7)	0.0008 (8)
N1	0.0292 (9)	0.0234 (8)	0.0207 (8)	−0.0013 (6)	0.0022 (7)	−0.0005 (6)
N2	0.0375 (9)	0.0255 (8)	0.0238 (9)	−0.0042 (7)	0.0052 (7)	0.0021 (6)
N3	0.0311 (9)	0.0309 (9)	0.0189 (8)	0.0003 (7)	0.0026 (7)	0.0055 (6)
N4	0.0252 (8)	0.0274 (8)	0.0225 (8)	−0.0018 (6)	0.0010 (7)	−0.0027 (6)
C1	0.0323 (10)	0.0253 (10)	0.0257 (10)	−0.0041 (8)	0.0037 (8)	−0.0012 (8)
C2	0.0242 (9)	0.0244 (9)	0.0193 (9)	0.0017 (7)	0.0035 (7)	0.0005 (7)
C3	0.0248 (9)	0.0253 (9)	0.0211 (9)	0.0040 (7)	0.0042 (7)	0.0007 (7)
C4	0.0566 (15)	0.0412 (13)	0.0274 (12)	0.0027 (11)	0.0064 (10)	0.0114 (9)
C5	0.0399 (12)	0.0357 (11)	0.0332 (12)	−0.0078 (9)	0.0011 (10)	−0.0100 (9)
C6	0.0202 (9)	0.0367 (11)	0.0205 (10)	0.0047 (8)	0.0029 (7)	−0.0006 (8)
C7	0.0257 (10)	0.0274 (9)	0.0223 (10)	0.0003 (7)	0.0039 (8)	0.0000 (7)

### Geometric parameters (Å, º)

**Table:**

Mg1—O3^i^	2.0672 (17)	N3—C6	1.361 (3)
Mg1—O3	2.0672 (17)	N3—C3	1.383 (2)
Mg1—O4^i^	2.081 (2)	N3—C4	1.459 (3)
Mg1—O4	2.081 (2)	N4—C6	1.382 (3)
Mg1—N1^i^	2.2255 (19)	N4—C7	1.414 (3)
Mg1—N1	2.2255 (19)	N4—C5	1.472 (3)
O1—C6	1.238 (2)	C1—H1	0.9500
O2—C7	1.238 (2)	C2—C3	1.381 (3)
O3—H3A	0.875 (10)	C2—C7	1.416 (3)
O3—H3B	0.872 (10)	C4—H4A	0.9800
O4—H4D	0.873 (10)	C4—H4B	0.9800
O4—H4E	0.867 (10)	C4—H4C	0.9800
N1—C1	1.334 (3)	C5—H5A	0.9800
N1—C2	1.398 (2)	C5—H5B	0.9800
N2—C3	1.337 (2)	C5—H5C	0.9800
N2—C1	1.361 (3)		
			
O3^i^—Mg1—O3	180.00 (3)	C6—N4—C5	115.94 (16)
O3^i^—Mg1—O4^i^	92.02 (7)	C7—N4—C5	117.55 (16)
O3—Mg1—O4^i^	87.98 (7)	N1—C1—N2	116.97 (17)
O3^i^—Mg1—O4	87.98 (7)	N1—C1—H1	121.5
O3—Mg1—O4	92.02 (7)	N2—C1—H1	121.5
O4^i^—Mg1—O4	180.0	C3—C2—N1	107.34 (17)
O3^i^—Mg1—N1^i^	90.06 (6)	C3—C2—C7	120.60 (17)
O3—Mg1—N1^i^	89.94 (6)	N1—C2—C7	132.06 (17)
O4^i^—Mg1—N1^i^	88.69 (6)	N2—C3—C2	111.89 (17)
O4—Mg1—N1^i^	91.31 (6)	N2—C3—N3	125.57 (17)
O3^i^—Mg1—N1	89.94 (6)	C2—C3—N3	122.53 (17)
O3—Mg1—N1	90.06 (6)	N3—C4—H4A	109.5
O4^i^—Mg1—N1	91.31 (6)	N3—C4—H4B	109.5
O4—Mg1—N1	88.69 (6)	H4A—C4—H4B	109.5
N1^i^—Mg1—N1	180.0	N3—C4—H4C	109.5
Mg1—O3—H3A	135.0 (19)	H4A—C4—H4C	109.5
Mg1—O3—H3B	111.8 (19)	H4B—C4—H4C	109.5
H3A—O3—H3B	103 (3)	N4—C5—H5A	109.5
Mg1—O4—H4D	122 (2)	N4—C5—H5B	109.5
Mg1—O4—H4E	125.3 (19)	H5A—C5—H5B	109.5
H4D—O4—H4E	108 (3)	N4—C5—H5C	109.5
C1—N1—C2	102.20 (16)	H5A—C5—H5C	109.5
C1—N1—Mg1	119.32 (12)	H5B—C5—H5C	109.5
C2—N1—Mg1	138.26 (13)	O1—C6—N3	121.81 (19)
C3—N2—C1	101.59 (16)	O1—C6—N4	120.91 (19)
C6—N3—C3	119.72 (16)	N3—C6—N4	117.28 (17)
C6—N3—C4	120.00 (17)	O2—C7—N4	118.96 (17)
C3—N3—C4	120.28 (17)	O2—C7—C2	127.74 (18)
C6—N4—C7	126.42 (16)	N4—C7—C2	113.31 (17)

Symmetry code: (i) −*x*+1, −*y*, −*z*+1.

### Hydrogen-bond geometry (Å, º)

**Table:**

*D*—H···*A*	*D*—H	H···*A*	*D*···*A*	*D*—H···*A*
O3—H3*A*···N2^ii^	0.88 (1)	1.97 (1)	2.809 (3)	160 (3)
O3—H3*B*···O2^i^	0.87 (1)	1.80 (1)	2.668 (2)	173 (3)
O4—H4*E*···O1^iii^	0.87 (1)	1.93 (1)	2.780 (2)	168 (3)
O4—H4*D*···O1^iv^	0.87 (1)	1.96 (1)	2.829 (3)	178 (3)

Symmetry codes: (i) −*x*+1, −*y*, −*z*+1; (ii) *x*, −*y*+1/2, *z*+1/2; (iii) −*x*, −*y*, −*z*; (iv) *x*, *y*, *z*+1.
